# Factors Associated with Smoking Status and Self-Reported Physical Activity Among Spanish Nursing Professionals: A Cross-Sectional Study

**DOI:** 10.3390/nursrep16080287

**Published:** 2026-08-18

**Authors:** Jose-Manuel Cuervo-Menéndez, Marta Sánchez-Zaballos, María-Carmen Migoya-Méndez, Sara Franco-Correia, Aida Gámez-Fernández, Maria-Pilar Mosteiro-Diaz

**Affiliations:** 1 Nursing Area, Department of Medicine, University of Oviedo, 33003 Oviedo, Spain; cuervomanuel@uniovi.es (J.-M.C.-M.); sanchezzmarta@uniovi.es (M.S.-Z.); francosara@uniovi.es (S.F.-C.); mmosteirod@uniovi.es (M.-P.M.-D.); 2 Sacyl Health Emergency Management, Castilla y León Health Service (Sacyl), 47007 Valladolid, Spain; uo183496@uniovi.es

**Keywords:** nurses, anxiety, depression, smoking, exercise, risk factors

## Abstract

**Background**: Modifiable lifestyle behaviours, including physical activity and smoking, are important determinants of health and may influence the mental well-being of nursing professionals. However, evidence simultaneously examining these factors and their associations with sociodemographic, occupational, and mental health factors remains limited. **Objectives**: The aim of this study was to analyse the sociodemographic and occupational factors, as well as anxiety and depressive symptoms, associated with smoking status and physical activity among Spanish nursing professionals. **Methods**: A cross-sectional study was conducted between June and August 2021, including 1216 Spanish nursing professionals. Participants completed an online questionnaire assessing sociodemographic and occupational characteristics, smoking status, physical activity, and symptoms of anxiety and depression using the Hospital Anxiety and Depression Scale (HADS). Bivariate analyses and multivariable logistic regression models identified factors associated with smoking status and physical activity. **Results**: Overall, 19.0% of participants were current smokers and 49.2% reported physical activity. Self-reported physical activity was positively associated with being a registered nurse, being male, being separated or widowed, living alone, and longer time working in the current service, whereas having children and current smoking were associated with lower odds of being physically active. Current smoking was less likely among older professionals, registered nurses, participants reporting physical activity, and those with anxiety symptoms. **Conclusions**: Physical activity and smoking showed an inverse association and were related to different sociodemographic and occupational characteristics among Spanish nursing professionals. The inverse association between anxiety symptoms and current smoking was unexpected and should be interpreted with caution. These findings may inform workplace health promotion strategies addressing physical activity and tobacco use together, while considering the specific needs of different professional groups within the nursing workforce.

## 1. Introduction

Modifiable behavioural risk factors are key determinants of health and represent important targets for disease prevention and health promotion [1]. In this context, the World Health Organization highlights the need to promote physical activity, reduce sedentary behaviour, and decrease tobacco use as core public health priorities [2,3]. The workplace is a particularly relevant setting for addressing these behaviours because it provides access to large populations and allows health promotion strategies to be integrated into the working environment [4]. Recent evidence suggests that workplace health promotion programmes can improve health-related practices, including increasing physical activity and supporting smoking cessation, although their effectiveness varies depending on the intervention and further high-quality research is needed [5,6].

This approach is especially relevant for nursing professionals, whose health-related behaviours may be influenced not only by individual choices but also by specific working conditions. Shift work, heavy workloads, and emotional demands are common in nursing and may hinder the adoption and maintenance of healthy lifestyle habits, potentially affecting both professionals’ well-being and the quality of patient care [7,8]. At the same time, mental health problems are highly prevalent among nurses and constitute a major occupational health challenge worldwide [9,10]: longitudinal studies have shown that direct exposure to infected patients, night shift work, and long working hours are associated with higher levels of anxiety, depression, and stress [9,10,11]. Much of this evidence was generated during the COVID-19 pandemic [11], a period in which these occupational demands were particularly intense, and which corresponds to the context in which the data for the present study were collected.

The Job Demands–Resources (JD-R) model provides a useful framework for understanding these relationships. According to this model, employee well-being results from the balance between job demands, which require sustained physical and psychological effort, and job resources, which support goal achievement and help buffer the impact of demands [12,13]. Two complementary processes are described: a health impairment process, through which chronic demands deplete energy and contribute to strain, and a motivational process, through which resources foster engagement and well-being. In nursing, night and rotating shift work, high workloads, time pressure, and emotional demands operate as job demands, whereas professional autonomy, supervisory support, and control over working time operate as job resources [7,14,15].

Applied to the present study, this framework provides a way of understanding smoking status and self-reported physical activity as health-related behaviours that may be shaped by the working environment rather than as isolated individual choices. Symptoms of anxiety and depression may be understood as indicators of strain that may be associated with both behaviours. Within this conceptual structure, previous research has established three sets of findings. First, physical activity is consistently associated with lower levels of anxiety, depression, and burnout, both in the general population [16] and among healthcare and nursing professionals [17,18]. Second, smoking is associated with a higher prevalence of anxiety and depressive symptoms, in a relationship that is likely to be bidirectional [19], and smoking cessation does not worsen, and may improve, psychological well-being [20]. Third, occupational exposures and workplace characteristics are associated with poorer mental health among nurses [10,11].

However, these associations have often been examined separately. Previous studies have typically focused on a single behaviour [16,17,18,19,20,21], occupational conditions have frequently been described in relation to mental health rather than analysed as determinants of health-related behaviours [10,11], and much of the available evidence derives from the general population [16,19,20]. As a result, it remains unclear whether the associations between mental health symptoms and health-related behaviours persist once occupational conditions are taken into account.

Nursing professionals constitute a distinctive occupational group in this respect. Their working conditions, including shift work, heavy workloads, and high emotional demands, are largely determined by the organisation of health services rather than individually chosen, and have been shown to hinder the adoption of healthy lifestyle habits and to be associated with poorer mental health [8,9,10,11]. Previous studies conducted by our research group have reported a high prevalence of anxiety and depressive symptoms, assessed using the Hospital Anxiety and Depression Scale (HADS), among both nursing students and Spanish registered nurses [22,23]. However, to the best of our knowledge, no previous study has comprehensively examined the associations of smoking status and physical activity with sociodemographic and occupational characteristics and symptoms of anxiety and depression in a sample of nursing professionals, including registered nurses and nursing assistants. Because these occupational conditions are potentially modifiable at the organisational level, identifying the factors associated with these behaviours may inform workplace health promotion strategies in a way that findings obtained in the general population cannot [6,7].

Based on the available evidence and the JD-R model, we hypothesised that smoking status and self-reported physical activity would not be associated only with sociodemographic characteristics, but also with occupational factors and symptoms of anxiety and depression. Therefore, the aim of this study was to analyse the sociodemographic and occupational factors, as well as anxiety and depressive symptoms, associated with smoking status and physical activity among Spanish nursing professionals.

## 2. Materials and Methods

### 2.1. Study Design

A cross-sectional study was conducted between June and August 2021. The study is reported in accordance with the Strengthening the Reporting of Observational Studies in Epidemiology (STROBE) Statement for cross-sectional studies [24]. The completed STROBE checklist is provided as Supplementary Materials.

### 2.2. Participants

The study population comprised nursing professionals working in Spain. For the purposes of this study, the term “nursing professionals” refers collectively to two occupational categories within the Spanish healthcare system: registered nurses and nursing assistants. Participants were eligible if they were registered nurses or nursing assistants, were actively working at the time of data collection, and voluntarily agreed to participate. Because active employment was an inclusion criterion, professionals who were on sick leave, maternity or paternity leave, or otherwise temporarily absent from work at the time of data collection were not eligible. No restrictions were applied regarding the clinical setting, unit, or position held.

### 2.3. Sample Size and Model Adequacy

The minimum required sample size was estimated a priori at 365 participants, assuming a 95% confidence level, a 5% margin of error, and maximum variability of the expected proportion. This calculation was performed to ensure adequate precision for the descriptive estimates of the study. Because the main analytical objective involved multivariable logistic regression models, the adequacy of the final analytical sample for these models was also assessed. No formal a priori power calculation was conducted specifically for the multivariable logistic regression models. Instead, the number of outcome events was examined in relation to the number of estimated model parameters after dummy coding categorical variables. Each multivariable logistic regression model included 18 estimated parameters. In the complete-case analyses, 1191 participants were included in each model. The physical activity model included 588 outcome events, corresponding to 32.7 events per estimated parameter, whereas the smoking status model included 224 outcome events, corresponding to 12.4 events per estimated parameter. Therefore, the final analytical sample was considered adequate for the planned multivariable logistic regression analyses.

### 2.4. Variables and Instruments

An online questionnaire was specifically designed for the study. It included sociodemographic variables, such as gender, age, marital status, children, and living arrangements; occupational variables, including years of professional experience, years of experience in the current service, professional category, type of work shift, and type of employment contract; and modifiable lifestyle-related variables, including smoking status and physical activity. Selected occupational variables were considered proxy indicators of job demands, while anxiety and depressive symptoms were considered indicators of psychological strain, in line with the Job Demands–Resources (JD-R) framework.

Smoking status was assessed as a dichotomous variable according to whether participants reported being current smokers or non-smokers. The non-smoker category therefore included both never smokers and former smokers, as the questionnaire did not distinguish between these two groups.

Physical activity was also assessed as a dichotomous variable according to whether participants reported engaging in physical exercise or sports. This measure captured participation in physical activity as a behaviour, rather than its frequency, duration, volume, or intensity.

Symptoms of anxiety and depression were assessed using the Hospital Anxiety and Depression Scale (HADS), developed by Zigmond and Snaith [25] and validated for the Spanish population by Herrero et al. [26]. The HADS is a widely used self-administered instrument designed to assess anxiety and depressive symptoms. It consists of 14 items divided into two subscales: the anxiety subscale (HADS-A) and the depression subscale (HADS-D), with seven items each. Each item is scored on a four-point Likert scale ranging from 0 to 3, resulting in a total score from 0 to 21 for each subscale. Higher scores indicate greater symptom severity. In the Spanish validation study, the HADS demonstrated good internal consistency, with a Cronbach’s alpha of 0.90 for the total scale, 0.85 for the anxiety subscale, and 0.84 for the depression subscale [26]. In the present sample, Cronbach’s alpha was 0.858 for the anxiety subscale and 0.775 for the depression subscale.

HADS subscale scores were described as continuous variables and then dichotomised using a cut-off point of ≥8 for bivariate and multivariable analyses. Scores ≥ 8 on each subscale are commonly used as a screening threshold for possible anxiety or depressive symptoms. This approach was chosen to facilitate the identification and interpretation of participants with possible symptoms in the multivariable analyses. The cut-off is intended for screening purposes and does not constitute a clinical diagnosis.

### 2.5. Data Collection

The online questionnaire was distributed using snowball sampling. Dissemination was carried out through official nursing associations, which shared the survey with their members through institutional mailing lists, as well as through social media platforms, including Telegram (https://telegram.org), WhatsApp (https://www.whatsapp.com), and Instagram (https://www.instagram.com), and through professional contacts. Because the survey was distributed through multiple open and indirect channels, the total number of nursing professionals who received the invitation could not be determined. Therefore, the response rate could not be calculated. The questionnaire remained available for two months. The first page of the online form included information about the study, its voluntary nature, and the confidential handling of data. Participants were required to confirm their voluntary consent by selecting an acceptance box before accessing the questionnaire. To reduce the risk of duplicate responses, the survey settings allowed only one submission per email address; this information was used solely for this technical purpose and removed before data analysis. No identifying information was included in the analysed database, and no section of the questionnaire collected personal data that would allow participants to be identified.

### 2.6. Data Analysis

Descriptive and exploratory analyses were first performed to summarise the characteristics of the study sample and to assess the distribution of continuous variables. Categorical variables were expressed as frequencies and percentages, whereas continuous variables were described using means and standard deviations for descriptive purposes and medians and interquartile ranges when appropriate. As continuous variables did not follow a normal distribution, between-group comparisons were performed using the Mann–Whitney U test. Two separate bivariate analyses were then performed: one according to smoking status and another according to physical activity. Participants were classified as smokers or non-smokers according to their self-reported smoking status, and as physically active or inactive according to whether they reported engaging in physical exercise or sports. Bivariate associations between sociodemographic, occupational, and mental health variables and each grouping variable were examined separately. For categorical variables, comparisons were performed using the chi-square test or Fisher’s exact test when appropriate. Anxiety and depressive symptoms, assessed using the HADS-A and HADS-D subscales, were analysed as dichotomous variables using scores ≥ 8 on each subscale as the cut-off point. Missing data were not imputed. Descriptive and bivariate analyses were performed using the available valid data for each variable. For the multivariable logistic regression models, complete-case analysis was applied; therefore, participants with missing data in any of the variables included in the corresponding model were excluded from that analysis. In both regression models, 1191 participants were included and 25 cases were excluded because of missing data. Two multivariable binary logistic regression models were fitted to identify factors independently associated with smoking status and physical activity. As part of the model-building process, multicollinearity among explanatory variables was assessed using tolerance and variance inflation factor (VIF) values obtained from equivalent linear regression models including the same set of predictors. Tolerance values < 0.10 and VIF values > 10 were considered indicative of problematic multicollinearity. Variables were selected a priori based on the study objective, previous evidence, and their conceptual relevance to smoking and physical activity among nursing professionals, rather than on bivariate statistical significance alone. Sociodemographic variables, occupational characteristics, anxiety and depressive symptoms, and the other lifestyle-related outcome were entered simultaneously into each model. Categorical predictors were dummy coded using indicator contrasts, with reference categories defined a priori according to conceptual relevance and interpretability. The number of explanatory variables included in each model was determined by this predefined conceptual model. Interaction terms were not included in the final models because no specific a priori interaction hypotheses were defined. The analyses therefore focused on main effects, in line with the explanatory objective of the study and to avoid unnecessary model complexity. Model discrimination was assessed using the area under the receiver operating characteristic curve (AUC), based on the predicted probabilities from each final logistic regression model. All statistical analyses were performed using IBM SPSS Statistics, version 30.0 (IBM Corp., Armonk, NY, USA).

### 2.7. Ethical and Legal Considerations

The study was conducted in accordance with the ethical principles of the Declaration of Helsinki [27] and complied with Organic Law 3/2018, of 5 December, on Personal Data Protection and Guarantee of Digital Rights [28]. Ethical approval was obtained from the Research Ethics Committee in Asturias, and all participants provided electronic informed consent before completing the questionnaire.

## 3. Results

### Participant Flow and Characteristics

A total of 1216 submitted questionnaires were available in the database, met the inclusion criteria, and were included in the descriptive analyses. Bivariate analyses were performed using the available valid data for each variable. For the multivariable logistic regression models, complete-case analysis was applied; therefore, 1191 participants were included and 25 were excluded because of missing data in at least one variable included in the models. The study sample comprised 979 registered nurses (80.5%) and 237 nursing assistants (19.5%). Participants were predominantly female (90.3%), with a mean age of 36.98 ± 11.32 years, and most were married (64.6%). Mean professional experience was 12.69 ± 10.73 years, while the mean time working in the current service was 6.14 ± 7.51 years. Overall, 63.9% worked rotating shifts, 19.0% were current smokers, and 49.2% reported physical activity. Anxiety symptoms (HADS-A ≥ 8) were identified in 61.1% of participants, and depressive symptoms (HADS-D ≥ 8) were present in 47.9%. Additional participant characteristics are presented in Table 1.

In the bivariate analysis, professional category, marital status, living arrangement, employment status, and household dependence on the participant’s income were significantly associated with smoking status (all *p* < 0.05), whereas no significant associations were observed for gender, having children, anxiety symptoms or depressive symptoms. Regarding self-reported physical activity, significant associations were observed for professional category, gender, living arrangement, having children, and depressive symptoms (all *p* < 0.05), whereas no significant associations were found for age, professional experience, years in the current service, marital status, employment status, household dependence on the participant’s income or anxiety symptoms (Table 2).

In the multivariable logistic regression model examining factors associated with self-reported physical activity, being a registered nurse (OR = 1.72, 95% CI: 1.23–2.41), being male (OR = 1.87, 95% CI: 1.23–2.82), longer time working in the current service/unit (OR = 1.03, 95% CI: 1.00–1.05), being separated or widowed compared with being single (OR = 3.10, 95% CI: 1.58–6.11), and living alone compared with living with parents (OR = 1.74, 95% CI: 1.08–2.82) were independently associated with higher odds of reporting physical activity. Conversely, having children (OR = 0.51, 95% CI: 0.35–0.73) and current smoking (OR = 0.68, 95% CI: 0.50–0.92) were associated with lower odds of reporting physical activity. Anxiety and depressive symptoms were not independently associated with physical activity in the adjusted model (Table 3).

In the multivariable logistic regression model examining factors associated with smoking status, being a registered nurse (OR = 0.42, 95% CI: 0.29–0.61), older age (OR = 0.96, 95% CI: 0.93–0.99), engaging in physical activity (OR = 0.68, 95% CI: 0.49–0.92), and anxiety symptoms (OR = 0.60, 95% CI: 0.41–0.88) were independently associated with lower odds of current smoking. Although separated or widowed participants showed higher odds of current smoking than single participants (OR = 2.17, 95% CI: 1.02–4.61; *p* = 0.044), the overall marital status variable did not reach statistical significance (*p* = 0.091) (Table 4).

No evidence of problematic multicollinearity was observed in either multivariable logistic regression model, according to the predefined criteria of tolerance < 0.10 or VIF > 10. In both models, the maximum VIF was 7.49 and the minimum tolerance value was 0.13. The physical activity model showed modest discrimination, with an AUC of 0.642 (95% CI: 0.610–0.673), whereas the smoking status model also showed modest discrimination, with an AUC of 0.649 (95% CI: 0.610–0.689).

## 4. Discussion

The present study identified several factors independently associated with self-reported physical activity and current smoking among Spanish nursing professionals. Physical activity and smoking showed an inverse association, suggesting that these modifiable lifestyle behaviours may be interrelated within this professional group rather than occurring in isolation. Professional category was associated with both outcomes: registered nurses had higher odds of reporting physical activity and lower odds of current smoking than nursing assistants. Other factors, including gender, age, marital status, living arrangements, having children, and years working in the current service, were associated with one of the two outcomes. Mental health-related findings were less consistent: anxiety symptoms were independently associated with lower odds of current smoking, whereas neither anxiety nor depressive symptoms were independently associated with physical activity after adjustment. From the perspective of the Job Demands–Resources (JD-R) framework, these findings suggest that health-related behaviours among nursing professionals should be interpreted not only as individual lifestyle choices, but also in relation to occupational context and psychological strain.

Regarding the relationship between mental health and physical activity, nursing professionals reporting depressive symptoms were more likely to report physical activity than those without symptoms (53.4% vs. 45.3%). However, this crude association was reversed and lost statistical significance after adjustment for sociodemographic and occupational characteristics (OR = 0.83, 95% CI: 0.62–1.12, *p* = 0.225), suggesting that depressive symptoms were not independently associated with self-reported physical activity once these factors were considered. These results should be interpreted alongside previous evidence showing that physical activity is generally associated with better mental health outcomes in the general population and among healthcare professionals [29,30]. Evidence from studies conducted in nursing professionals also supports this relationship. Lower levels of depression and stress have been associated with moderate- and vigorous-intensity physical activity, while exercise interventions have demonstrated beneficial effects on psychological well-being and sleep quality [31,32]. In addition, depressive symptoms have been identified as one of the main factors associated with poorer quality of working life among nurses [31,32,33]. Taken together, this evidence does not contradict the present findings but suggests that, in this sample, the crude association between depressive symptoms and physical activity may be explained by other correlated factors (such as professional category, gender, or family responsibilities) rather than reflecting an independent relationship. Overall, this evidence supports workplace strategies that facilitate physical activity as part of broader efforts to improve mental health and occupational well-being in nursing professionals.

Another relevant finding was the inverse relationship between current smoking and self-reported physical activity. Physical activity was linked to a lower likelihood of current smoking, while current smoking was related to lower engagement in physical activity in the multivariable models. The consistency of this association across both multivariable models supports the presence of an inverse association between these two lifestyle behaviours. These findings are in line with previous evidence suggesting that healthy lifestyle habits tend to cluster rather than occur in isolation. Zhang et al. [34] reported that recreational physical activity was independently associated with a lower likelihood of smoking, whereas sedentary behaviour was positively associated with tobacco use. Similarly, Moghimi et al. [35] found that nursing professionals with healthier lifestyles reported higher levels of physical activity together with other health-promoting practices, supporting the concept that healthy lifestyle choices may coexist rather than occur in isolation.

With respect to smoking behaviour, anxiety symptoms did not show a significant association in the bivariate analysis (*p* = 0.139) but were independently associated with lower odds of current smoking in the multivariable model (*p* = 0.009). This unexpected finding after adjustment may reflect residual confounding or a suppression effect, although the specific mechanism cannot be established from the present cross-sectional data [36]. This finding contrasts with previous evidence among nursing professionals, including the positive association between anxiety and smoking reported by Asad et al. [21]. However, Taylor et al. [20] reported improvements in anxiety symptoms following smoking cessation, suggesting that the relationship between smoking and anxiety may be complex and potentially bidirectional. More broadly, evidence from the general population has also linked smoking with mental health outcomes [36,37,38]. Differences in study design, populations, measurement of anxiety, and adjustment for potential confounding factors may partly explain the discrepancies between studies. Given the cross-sectional design of the present study, the direction of the observed association cannot be established, and reverse causality and residual confounding cannot be excluded. Longitudinal studies are therefore needed to clarify the temporal relationship between anxiety and smoking among nursing professionals. Depressive symptoms were not independently associated with smoking after adjustment.

The prevalence of anxiety symptoms (61.1%) and depressive symptoms (47.9%) observed in this study was high. These findings should be interpreted in the context of data collection during the COVID-19 pandemic, which had a substantial impact on the mental health of healthcare professionals. A systematic review of Spanish healthcare workers during the COVID-19 pandemic reported pooled prevalences of 35.25% for moderate-to-severe anxiety symptoms and 29.76% for moderate-to-severe depressive symptoms [39]. Similarly, a cross-sectional study of Spanish nurses found that 68% experienced some level of depression, anxiety, insomnia, or distress, with 38% reporting moderate or severe symptoms [40]. However, comparisons should be made cautiously because of differences in study populations, measures, and definitions of psychological symptoms. In particular, the pooled estimates cited above refer to moderate-to-severe symptoms, whereas the HADS cut-off of ≥8 applied here also captures possible symptomatology, which largely accounts for the higher figures observed in the present study. In addition, this threshold is a screening rather than a diagnostic criterion, and the volunteer-based snowball sampling may also have influenced the observed prevalence.

Professional category was independently associated with health-related behaviours. Registered nurses showed higher odds of engaging in physical activity and lower odds of current smoking than nursing assistants. Although evidence directly comparing these professional groups is limited, differences in occupational demands may partly explain these findings. Nursing assistants accumulated higher levels of occupational physical activity than registered nurses during working shifts, whereas leisure-time physical activity was similar between the two groups [41]. Furthermore, a systematic review of healthcare workers concluded that occupational and leisure-time physical activity are distinct constructs that differ in intensity, duration, and health effects, and therefore should be considered separately when evaluating physical activity behaviours [42]. Alternative explanations nevertheless warrant consideration. Differences in educational level, health literacy, and socioeconomic position across professional categories may contribute to the observed patterns: lower educational attainment has been consistently associated with greater exposure to unhealthy lifestyle factors, including smoking and physical inactivity [43], whereas higher educational attainment has been linked to a greater likelihood of adopting healthy lifestyles [44]. Health literacy has likewise been independently associated with health-related lifestyle behaviours in health-management professionals [45]. In addition, registered nurses may benefit from greater professional autonomy and control over their work environment, a recognised component of healthy work environments that has been associated with nurses’ well-being and job satisfaction [46,47]. Given the cross-sectional design of the present study, these interpretations remain tentative, and the direction of the observed associations cannot be established.

Several sociodemographic and occupational characteristics were independently associated with physical activity. Male gender, being a registered nurse, and longer time working in the current service were associated with greater engagement in physical activity, whereas having children was associated with a lower likelihood of being physically active. The association between longer service tenure and higher physical activity may reflect that professionals with greater tenure have generally attained more predictable work schedules and greater familiarity with unit routines, conditions that favour the consolidation of stable personal habits, including regular exercise [48]; conversely, staff recently incorporated into a service may prioritise adaptation to their new role and shift arrangements over leisure-time activity. Although this association was statistically significant, its magnitude was modest (OR = 1.03 per year), indicating that its practical relevance is limited and that tenure alone should not be regarded as a meaningful determinant of physical activity engagement. Separated or widowed professionals showed markedly higher odds of engaging in physical activity, and living alone was likewise associated with greater physical activity.

Existing evidence has also linked physical activity with several personal and occupational characteristics among healthcare professionals, although not always in the same direction. Older age and greater professional experience have been positively correlated with physical activity among emergency and outpatient nurses [49] and moderate physical activity has also been reported to be more common among older nurses [50]. In the present sample, however, neither age nor total professional experience was independently associated with physical activity; only length of service in the current unit was, which may suggest that occupational stability, rather than age or career length per se, may partly underlie this association. Lifestyle patterns have also been reported to differ by gender among healthcare workers [50,51] and female healthcare workers report significantly less leisure time than their male counterparts and cite lack of time as a barrier to exercise more frequently [52] which is consistent with the higher odds of physical activity observed among men in this study. Although evidence on the influence of family responsibilities remains limited, childcare demands and work–life balance constraints may partly account for the lower likelihood of engaging in physical activity among professionals with children [53]. Conversely, the higher levels of physical activity observed among separated or widowed professionals and among those living alone may reflect greater availability of discretionary time outside working hours, since lack of time is the most frequently reported reason for physical inactivity among healthcare workers [52]. These findings should nevertheless be interpreted with caution, given the small number of separated or widowed participants *(n* = 68) and the resulting wide confidence interval, as well as the cross-sectional design, which precludes any inference about the direction of the observed associations.

From a practical perspective, the present findings suggest that improving the occupational health of nursing professionals requires integrated strategies that simultaneously address health-related behaviours, mental health, and occupational conditions, prioritising multicomponent and multilevel interventions over isolated actions [54,55,56]. In this regard, occupational health services and nursing managers could consider multicomponent interventions combining opportunities for physical activity, smoking cessation support, psychological well-being, and organisational measures aimed at reducing excessive job demands and strengthening job resources. Examples include brief exercise or walking programmes, structured rest breaks, leadership training for nurse managers, digital modules targeting stress management and resilience, and brief smoking cessation counselling integrated into routine occupational health check-ups [54,55,56]. More recently, integrated programmes combining work-directed strategies, such as workload redistribution and healthy work design, with person-directed components have emerged as a promising and scalable approach for supporting nurses’ well-being [56]. Given the heterogeneity of existing evidence, these strategies should be adapted to each healthcare organisation, developed with the active involvement of nursing professionals, and regularly evaluated [54,55,56].

Some limitations should be considered when interpreting these findings. First, although the JD-R model was used as a conceptual framework, only proxy indicators of job demands were available, including shift pattern, employment status, professional category, and length of service. Job resources were not directly assessed; therefore, the model was only partially operationalised in the present study. Second, the cross-sectional design precludes establishing causal relationships or determining the direction of the associations observed. Reverse causality cannot be ruled out, since physical activity, smoking, and mental health symptoms may influence one another in both directions. Third, participants were recruited using a snowball sampling strategy and participation was voluntary, which may have introduced selection bias. Professionals with a greater interest in healthy lifestyles or mental health may have been more likely to participate, potentially affecting prevalence estimates. As recruitment was open and web-based, a response rate could not be calculated. Although the sample included registered nurses and nursing assistants from different settings across Spain, it cannot be assumed to be fully representative of the Spanish nursing workforce. Although the analyses were adjusted for several sociodemographic, occupational, and mental health variables, residual confounding cannot be ruled out. Other potentially relevant factors not assessed in the present study, such as dietary habits, alcohol consumption, sleep quality, chronic health conditions, or organisational factors, may also have influenced the observed associations. Fourth, all variables were self-reported, so social desirability and recall bias cannot be excluded, particularly for smoking and physical activity. In addition, smoking status and physical activity were assessed using single-item self-reported measures, which may have resulted in some degree of misclassification. Validated multi-item instruments were not administered because the questionnaire had to cover a broad set of variables and its length was restricted to limit respondent burden. Physical activity was assessed as a dichotomous variable and did not capture frequency, intensity, duration, or domain of activity, or adherence to the World Health Organization physical activity recommendations, while smoking status did not differentiate between former smokers, never smokers, frequency of smoking, or level of tobacco consumption. The non-smoking category therefore included both never smokers and former smokers, two groups that differ in lifestyle and health characteristics, which may have diluted the contrast between categories. Any misclassification of this kind would most likely attenuate the observed associations rather than create spurious ones. Therefore, these findings should be interpreted as reflecting broad indicators of lifestyle behaviours rather than comprehensive measures of physical activity or tobacco use. Finally, anxiety and depressive symptoms were assessed using the HADS as a screening instrument rather than through a clinical diagnostic interview. These aspects should be taken into account when comparing the present findings with studies using more detailed lifestyle or mental health measures. The discriminative capacity of both models was also moderate, suggesting that relevant factors associated with these behaviours were not captured by the models. Caution should also be exercised when applying these results to healthcare systems with different organisational, cultural, or occupational characteristics. Future studies using probability-based sampling strategies and more detailed measures of lifestyle behaviours would help confirm and extend these findings.

Despite these limitations, the present study has several strengths. It included a large sample of Spanish nursing professionals and incorporated both registered nurses and nursing assistants, allowing lifestyle-related factors to be examined across different professional categories within the nursing workforce. In addition, smoking status and physical activity were analysed simultaneously, providing a more integrated perspective on modifiable lifestyle practices rather than considering them as isolated factors. The inclusion of sociodemographic, occupational, lifestyle, and mental health-related variables in the same analytical models also allowed a broader assessment of the factors associated with each outcome. Moreover, anxiety and depressive symptoms were assessed using the HADS, a widely used screening instrument, which facilitates comparison with previous research in healthcare professionals.

## 5. Conclusions

This study provides an exploratory integrated analysis of two modifiable lifestyle practices, physical activity and tobacco use, among Spanish nursing professionals. The findings suggest that these behaviours are not isolated but are related to sociodemographic and occupational characteristics, with an inverse association observed between physical activity and smoking. Physical activity was mainly associated with professional category, gender, family structure, and length of service in the current unit, whereas current smoking was less likely among registered nurses, older professionals, those reporting physical activity, and those with anxiety symptoms. The inverse association between anxiety symptoms and current smoking was unexpected and should be interpreted with caution, as it contrasts with much of the previous literature and requires confirmation in longitudinal studies. Depressive symptoms were not independently associated with either physical activity or smoking status.

These findings may inform workplace health promotion strategies aimed at addressing physical activity and tobacco use together while considering the specific needs of different professional groups within the nursing workforce. However, the cross-sectional design, the use of simplified lifestyle behaviour measures, and the non-probability sampling strategy warrant caution in interpreting these results. Future longitudinal studies using more comprehensive behavioural assessments are needed to clarify the direction of these associations and to inform the development of tailored preventive interventions for nursing professionals.

## Figures and Tables

**Table 1:** Sociodemographic, occupational, lifestyle and mental health characteristics of the study participants (*N* = 1216).

Variable	Category	*n*	%
**Sociodemographic characteristics**			
**Gender**	Woman	1098	90.3
	Man	118	9.7
**Age (years)**		Mean 36.98	SD 11.32
**Marital status**	Married	786	64.6
	Single	351	28.9
	Separated/Widowed	73	6.0
**Children**	Yes	486	40.0
	No	730	60.0
**Household depends exclusively on participant’s income**	Yes	230	18.9
	No	986	81.1
**Occupational characteristics**			
**Professional category**	Registered nurse	979	80.5
	Nursing assistant	237	19.5
**Professional experience (years)**	Mean 12.69	SD 10.73	
**Time working in current service (years)**	Mean 6.14	SD 7.51	
**Employment status**	Temporary contract	530	43.6
	Replacement contract	371	30.5
	Permanent position	315	25.9
**Shift pattern**	Fixed shift	439	36.1
	Rotating shift	777	63.9
**Lifestyle behaviours**			
**Smoking status**	Yes	231	19.0
	No	985	81.0
**Physical activity**	Yes	597	49.2
	No	617	50.8
**Mental health (HADS)**			
**Anxiety symptoms (HADS-A)**, mean ± SD		8.61 ± 4.01	
Anxiety symptoms (HADS-A ≥ 8)	No	473	38.9
	Yes	743	61.1
**Depression symptoms (HADS-D)**, mean ± SD		7.48 ± 3.87	
Depression symptoms (HADS-D ≥ 8)	No	634	52.1
	Yes	582	47.9

Abbreviations: HADS, Hospital Anxiety and Depression Scale; HADS-A, Hospital Anxiety and Depression Scale–Anxiety subscale; HADS-D, Hospital Anxiety and Depression Scale–Depression subscale; SD, standard deviation.

**Table 2:** Bivariate analysis according to smoking status and self-reported physical activity.

		Smoking Status			Physical Activity		
Variable	Category	Non-Smoker	Current Smoker	*p*-Value	Non-Active	Active	*p*-Value
**Age**		37.19 ± 11.47; 35 (27–45)	36.16 ± 10.67 35 (27–42)	0.314	37.21 ± 11.55 35 (27–45.5)	36.80 ± 11.10 35 (27–45)	0.592
**Professional experience**		12.76 ± 10.78 10 (3–20)	11.76 ± 10.04 8 (3–19)	0.472	12.44 ± 10.81 10 (3–20)	12.72 ± 10.49 10 (3–20)	0.564
**Years in current service**		6.14 ± 7.58 3 (1–10)	5.70 ± 6.86 3 (1–7)	0.580	5.73 ± 7.17 3 (1–8)	6.41 ± 7.73 3 (1–10)	0.189
**Professional category**	Registered nurse	819 (83.7)	160 (16.3)	<0.001	465 (47.6)	512 (52.4)	<0.001
	Nursing assistant	166 (70.0)	71 (30.0)		152 (64.1)	85 (35.9)	
**Marital status**	Married	651 (82.8)	135 (17.2)	0.003	409 (52.2)	375 (47.8)	0.633
	Single	282 (80.3)	69 (19.7)		171 (48.7)	180 (51.3)	
	Separated/Widowed	48 (65.8)	25 (34.2)		34 (46.6)	39 (53.4)	
**Gender**	Woman	885 (80.6)	213 (19.4)	0.275	576 (52.5)	522 (47.5)	<0.001
	Man	100 (84.7)	18 (15.3)		41 (35.3)	75 (64.7)	
**Living arrangement**	With partner and children	530 (83.5)	105 (16.5)	0.023	323 (50.9)	311 (49.1)	0.020
	With parents	163 (82.3)	35 (17.7)		104 (52.8)	93 (47.2)	
	Alone	133 (78.7)	36 (21.3)		69 (40.8)	100 (59.2)	
	Other	159 (74.3)	55 (25.7)		121 (56.5)	93 (43.5)	
**Children**	No	585 (80.1)	145 (19.9)	0.345	346 (47.5)	383 (52.5)	0.004
	Yes	400 (82.3)	86 (17.7)		271 (55.9)	214 (44.1)	
**Employment status**	Temporary	430 (81.1)	100 (18.9)	0.037	271 (51.3)	257 (48.7)	0.866
	Replacement	287 (77.4)	84 (22.6)		190 (51.2)	181 (48.8)	
	Permanent	268 (85.1)	47 (14.9)		156 (49.5)	159 (50.5)	
**Household depends exclusively on participant’s income**	No	810 (82.4)	173 (17.6)	0.021	494 (50.4)	487 (49.6)	0.206
	Yes	172 (74.8)	58 (25.2)		120 (52.2)	110 (47.8)	
**Anxiety symptoms (HADS-A)**	No	393 (83.1)	80 (16.9)	0.139	250 (53.1)	221 (46.9)	0.211
	Yes	592 (79.7)	151 (20.3)		367 (49.4)	376 (50.6)	
**Depressive symptoms (HADS-D)**	No	526 (83.0)	108 (17.0)	0.069	346 (54.7)	286 (45.3)	0.004
	Yes	459 (78.9)	123 (21.1)		271 (46.6)	311 (53.4)	

Abbreviations: HADS-A, Hospital Anxiety and Depression Scale–Anxiety subscale; HADS-D, Hospital Anxiety and Depression Scale–Depression subscale. Continuous variables are presented as mean ± SD and median (P25–P75), and categorical variables as *n* (%). *p*-values were obtained using the chi-square test for categorical variables and the Mann–Whitney U test for continuous variables.

**Table 3:** Multivariable analysis of factors associated with physical activity.

Variable	Category	OR (95% CI)	*p*
**Sociodemographic characteristics**			
**Gender**	Woman	–	
	Man	1.87 (1.23–2.82)	0.003
**Age (years)**		0.99 (0.96–1.01)	0.294
**Marital status**	Single	–	0.002
	Separated/Widowed	3.10 (1.58–6.11)	0.001
	Married	0.95 (0.66–1.38)	0.791
**Children**	No	–	
	Yes	0.51 (0.35–0.73)	<0.001
**Household depends exclusively on participant’s income**	No	–	
	Yes	0.70 (0.49–1.01)	0.058
**Living arrangement**	With parents	–	0.004
	Other	0.90 (0.58–1.41)	0.648
	Partner and children	1.55 (0.98–2.45)	0.062
	Alone	1.74 (1.08–2.82)	0.024
**Occupational characteristics**			
**Professional category**	Nursing assistant	–	
	Registered nurse	1.72 (1.23–2.41)	0.002
**Professional experience (years)**		1.01 (0.98–1.04)	0.605
**Years working in current service (years)**		1.03 (1.00–1.05)	0.022
**Employment status**	Temporary	–	0.880
	Replacement	1.05 (0.76–1.45)	0.767
	Permanent	0.97 (0.62–1.50)	0.874
**Shift pattern**	Fixed	–	
	Rotating	0.89 (0.68–1.15)	0.377
**Lifestyle behaviours**			
**Smoking status**	No	–	
	Yes	0.68 (0.50–0.92)	0.014
**Mental health (HADS)**			
**Anxiety symptoms (HADS-A)**	No	–	
	Yes	0.95 (0.70–1.28)	0.739
**Depressive symptoms (HADS-D)**	No	–	
	Yes	0.83 (0.62–1.12)	0.225

Abbreviations: OR, odds ratio; CI, confidence interval; HADS, Hospital Anxiety and Depression Scale; HADS-A, Hospital Anxiety and Depression Scale–Anxiety subscale; HADS-D, Hospital Anxiety and Depression Scale–Depression subscale. Model statistics: likelihood ratio χ^2^ = 77.53; Hosmer–Lemeshow test, *p* = 0.084; Nagelkerke R^2^ = 0.084; AUC = 0.642, 95% CI: 0.610–0.673.

**Table 4:** Multivariable analysis of factors associated with smoking status.

Variable	Category	OR (95% CI)	*p*
**Sociodemographic characteristics**			
**Gender**	Woman	–	
	Man	0.93 (0.54–1.61)	0.795
**Age (years)**		0.96 (0.93–0.99)	0.018
**Marital status**	Single	–	0.091
	Separated/Widowed	2.17 (1.02–4.61)	0.044
	Married	0.95 (0.60–1.49)	0.816
**Children**	No	–	
	Yes	0.83 (0.53–1.32)	0.434
**Household depends exclusively on participant’s income**	No	–	
	Yes	1.16 (0.76–1.78)	0.497
**Living arrangement**	With parents	–	0.359
	Other	1.64 (0.94–2.86)	0.079
	Partner and children	1.45 (0.81–2.61)	0.213
	Alone	1.47 (0.81–2.68)	0.208
**Occupational characteristics**			
**Professional category**	Nursing assistant	–	
	Registered nurse	0.42 (0.29–0.61)	<0.001
**Professional experience (years)**		1.01 (0.97–1.05)	0.560
**Years working in the current service (years)**		1.01 (0.98–1.05)	0.427
**Employment status**	Temporary	–	0.111
	Replacement	1.35 (0.91–1.99)	0.139
	Permanent	0.90 (0.52–1.57)	0.706
**Shift pattern**	Fixed	–	
	Rotating	0.90 (0.65–1.26)	0.542
**Lifestyle behaviours**			
**Physical activity**	No	–	
	Yes	0.68 (0.49–0.92)	0.014
**Mental health (HADS)**			
**Anxiety symptoms (HADS-A)**	No	–	
	Yes	0.60 (0.41–0.88)	0.009
**Depressive symptoms (HADS-D)**	No	–	
	Yes	1.23 (0.83–1.80)	0.303

Abbreviations: OR, odds ratio; CI, confidence interval; HADS, Hospital Anxiety and Depression Scale; HADS-A, Hospital Anxiety and Depression Scale–Anxiety subscale; HADS-D, Hospital Anxiety and Depression Scale–Depression subscale. Model statistics: likelihood ratio χ^2^ = 60.59; Hosmer–Lemeshow test, *p* = 0.445; Nagelkerke R^2^ = 0.080; AUC = 0.649, 95% CI: 0.610–0.689.

## Data Availability

The data presented in this study are not publicly available due to privacy and ethical restrictions.

## Supplementary Materials

The following supporting information can be downloaded at: https://www.mdpi.com/article/10.3390/nursrep16080287/s1, Supplementary File S1: Completed STROBE checklist for cross-sectional studies [24].

## Public Involvement Statement

No public involvement in any aspect of this research.

## Guidelines and Standards Statement

This manuscript was prepared in accordance with the Strengthening the Reporting of Observational Studies in Epidemiology (STROBE) Statement for cross-sectional studies.
