# Supplementary material for: Factors Associated with Smoking Status and Self-Reported Physical Activity Among Spanish Nursing Professionals: A Cross-Sectional Study
Source: Nurs Rep. 2026 Aug 18;16(8):287. doi: 10.3390/nursrep16080287 (PMC13516599; doi:10.3390/nursrep16080287)
Supplement: Supplementary file 1 [file nursrep-16-00287-s001.zip › nursrep-4466541-supplementary.pdf]

Supplementary File S1. Completed STROBE checklist for cross-sectional studies *Factors Associated with Smoking Status and Self-Reported Physical Activity among Spanish Nursing Professionals: A Cross-Sectional Study*

	Item No	Recommendation	Reported on page No / Section
**Title and abstract**	1	*(a)* Indicate the study's design with a commonly used term in the title or the abstract<br>*(b)* Provide in the abstract an informative and balanced summary of what was done and what was found	p. 1 (title: "A Cross-Sectional Study")<br>p. 1 (Abstract)
**Introduction**			
Background/rationale	2	Explain the scientific background and rationale for the investigation being reported	p. 1-3 (Introduction)
Objectives	3	State specific objectives, including any prespecified hypotheses	p. 3 (end of Introduction)
**Methods**			
Study design	4	Present key elements of study design early in the paper	p. 3 (Section 2.1 Study design)
Setting	5	Describe the setting, locations, and relevant dates, including periods of recruitment, exposure, follow-up, and data collection	p. 3 (2.1, study design); p.4-5 (2.5 Data collection)
Participants	6	*(a)* Give the eligibility criteria, and the sources and methods of selection of participants	p. 3 (2.2 Participants); p. 4-5 (2.5 Data collection – snowball sampling)
Variables	7	Clearly define all outcomes, exposures, predictors, potential confounders, and effect modifiers. Give diagnostic criteria, if applicable	p. 4 (2.4 Variables and instruments)
Data sources/ measurement	8*	For each variable of interest, give sources of data and details of methods of assessment (measurement). Describe comparability of assessment methods if there is more than one group	p. 4 (2.4, incl. HADS description and psychometrics)
Bias	9	Describe any efforts to address potential sources of bias	p. 4-5 (2.5 Data collection);<br>p. 16 (Discussion, limitations paragraph)
Study size	10	Explain how the study size was arrived at	p. 6–7 (2.3 Sample size and model adequacy)
Quantitative variables	11	Explain how quantitative variables were handled in the analyses. If applicable, describe which groupings were chosen and why	p. 7–8 (2.4, HADS-A/HADS-D dichotomised at ≥ 8)
Statistical methods	12	*(a)* Describe all statistical methods, including those used to control for confounding<br>*(b)* Describe any methods used to examine subgroups and interactions<br>*(c)* Explain how missing data were addressed	p. 5 (2.6 Data analysis)<br>p. 5 (interaction terms not included – stated and justified)<br>p. 5 (Data analysis)

	Item No	Recommendation	Reported on page No / Section
		(d) If applicable, describe analytical methods taking account of sampling strategy<br>*(e)* Describe any sensitivity analyses	Not applicable (non-probability, snowball sample; not addressed)<br>Not reported
**Results**			
Participants	13*	*(a)* Report numbers of individuals at each stage of study—eg numbers potentially eligible, examined for eligibility, confirmed eligible, included in the study, completing follow-up, and analysed<br>*(b)* Give reasons for non-participation at each stage<br>*(c)* Consider use of a flow diagram	p. 6 (3.1- Participant flow and characteristics)<br>p. 4-5 (2.5 Data collection: response rate could not be calculated – open, indirect distribution)<br>Not used – no flow diagram included
Descriptive data	14*	*(a)* Give characteristics of study participants (eg demographic, clinical, social) and information on exposures and potential confounders<br>*(b)* Indicate number of participants with missing data for each variable of interest	p. 10–12 (Table 1)<br>p. 10
Outcome data	15*	Report numbers of outcome events or summary measures	p. 7 (Table 1: smoking; physical activity)
Main results	16	*(a)* Give unadjusted estimates and, if applicable, confounder-adjusted estimates and their precision (eg, 95% confidence interval). Make clear which confounders were adjusted for and why they were included<br>*(b)* Report category boundaries when continuous variables were categorized<br>*(c)* If relevant, consider translating estimates of relative risk into absolute risk for a meaningful time period	p. 8-9 (Table 2, unadjusted bivariate); p. 10-12 (Tables 3–4, adjusted OR with 95% CI)<br>p. 4 (2.4 Variables and instruments)<br>Not applicable / not reported
Other analyses	17	Report other analyses done—eg analyses of subgroups and interactions, and sensitivity analyses	Not reported – no subgroup, interaction or sensitivity analyses performed
**Discussion**			
Key results	18	Summarise key results with reference to study objectives	p. 13 (Discussion, first paragraph)
Limitations	19	Discuss limitations of the study, taking into account sources of potential bias or imprecision. Discuss both direction and magnitude of any potential bias	p. 16 (Discussion, limitations paragraph)
Interpretation	20	Give a cautious overall interpretation of results considering objectives, limitations, multiplicity of analyses, results from similar studies, and other relevant evidence	p. 13-16 (Discussion)

	Item No	Recommendation	Reported on page No / Section
Generalisability	21	Discuss the generalisability (external validity) of the study results	p. 16 (Discussion, limitations paragraph)
**Other information**			
Funding	22	Give the source of funding and the role of the funders for the present study and, if applicable, for the original study on which the present article is based	p. 18 ("Funding: This research received no external funding")

This checklist was completed according to the STROBE Statement for cross-sectional studies.
